# Perception of Lexical Neutral Tone Among Adults and Infants

**DOI:** 10.3389/fpsyg.2018.00322

**Published:** 2018-03-23

**Authors:** Shanshan Fan, Aijun Li, Ao Chen

**Affiliations:** ^1^School of Preparatory Education, Beijing Language and Culture University, Beijing, China; ^2^Institute of Linguistics, Chinese Academy of Social Sciences, Beijing, China; ^3^School of Communication Science, Beijing Language and Culture University, Beijing, China; ^4^Utrecht Institute of Linguistics OTS, Utrecht University, Utrecht, Netherlands

**Keywords:** perceptual reorganization, tone acquisition, lexical neutral tone, lexical stress, cross-language comparison

## Abstract

Neutral tone (T0) is a special tone form in Mandarin that contains tonal and stress information. Compared with canonical tones, T0 has a much shorter duration and reduced pitch contour. Its tonal contour is determined by the preceding canonical tone. However, not much is known about the perception of tonal and stress information in T0. In the current study, we investigate (1) whether T0 can be perceived as lexically unstressed by stress-language listeners; and (2) how Mandarin (tone language)- and Dutch (stress language)-learning infants perceive T0. Three experiments were conducted. In Experiment 1, Dutch adults identified T0 as unstressed when presented with disyllabic sequences ending in T0. In Experiment 2, we used the visual fixation paradigm to test 4- to 6-month-old and 10- to 12-month-old Dutch and Mandarin infants on pseudoword discrimination (/pan1san4/ [high-level + high-falling] and /pan1san0/ [high-level + mid-falling]). T4 and T0 each exhibit a similar falling contour. The results show that (1) after being habituated to neutral tone sequences (/pan1san0/), Dutch infants discriminated the T1T0–T1T4 contrast; and (2) neither age groups of Mandarin infants discriminated the tone contrast. Assuming Mandarin infants’ lack of discrimination might be due to the similar F0 contours, we tested Mandarin infants in Experiment 3 using a more salient contrast, /pan1san2/ (high-level + mid-rising) and /pan1san0/. While no overall discrimination was observed, those who were habituated to /pan1san0/ demonstrated discrimination. The continuous discrimination of Dutch infants suggests that they might process neutral–canonical tone contrast as lexical stress rather than as tonal information. Overall, Mandarin infants’ failure implies that the representation of T0 is not complete during their 1st year of life; the acquisition of tonal categories may therefore take longer than we expected.

## Introduction

Lexical tones are pitch variations that distinguish lexical meanings. Mandarin is the most widely studied tone language, in which four canonical tones are used to distinguish word meanings, including T1 (high-level; 55 in Chao tone letters), T2 (mid-rising; 35), T3 (low-dipping; 21/214) and T4 (high-falling; 51). For example, the following words have different meanings based on canonical tones:/ma1/ (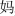, mother), /ma2/ (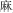, numb), /ma3/ (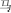, horse), and /ma4/ (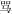, to scold). Besides the four canonical tones, neutral tone (T0) never occurs independently or at the beginning of a word. It is always preceded by a canonical tone. Neutral tone can distinguish word meanings, such as 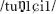
(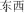, east and west) and 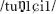 (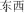, things), and appear in different lexical and syntactic contexts, including reduplication, affixation, lexeme type, directional complements, complement particles, etc. With regard to lexeme type, words are distinguished solely by the presence of neutral tone without any other morphological or grammatical marker, such as 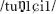 (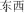, east and west) vs. 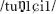 (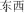, things) (Luo and Wang, 2002; Lin, 2012). In the present study, we focus on the lexeme type.

**FIGURE 1:**
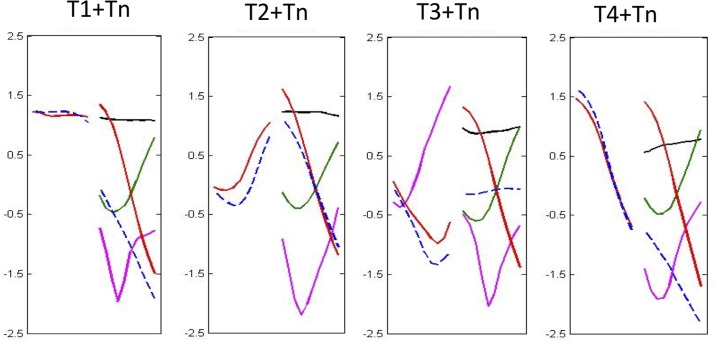
F0 contour patterns of all possible disyllabic tonal combinations (dashed lines represent neutral tone combinations). The vertical axis is the normalized *z*-score; the horizontal axis is the normalized duration (Li and Gao, 2017).

Neutral tone is acoustically light with a shorter duration and reduced pitch contour. It has been referred to as unstressed or weak stress in previous studies (Chao, 1979; Zhu, 2002; Lu and Wang, 2005; Wei, 2005; Duanmu, 2007; Cao, 2008; Deng, 2010; Jia, 2011; Bao and Lin, 2014). The tonal contour of T0 is determined by the preceding canonical tone. When preceded by T1, T2, or T4, the tonal contour of T0 is falling; when preceded by T3, the tonal contour is mid-level (Chao, 1979; Wu, 1992; Kong and Lü, 1998; Luo and Wang, 2002; Lin and Wang, 2013; Zhang and Li, 2016). Neutral tone has a lower pitch register and narrower pitch range. Pitch patterns are shown in **Figure 1**, where the dashed lines denote sequences ending with a neutral tone. The duration of neutral tone is about 50% of its corresponding canonical tone (Lin and Yan, 1980; Lin, 1983; Lee, 2003) or about 60% of the preceding canonical tone (Cao, 1986; Li, 2017). In summary, neutral tone contrasts with canonical tone lexically because the neutral tone is unstressed and has distinguished pitch pattern. Neutral tone possesses properties of lexical stress and lexical tone.

The acoustic correlates of neutral tone are duration, F0, intensity, and spectral features (i.e., vowel reduction, initial consonant voicing, and spectral tilt steeping). The main acoustic correlates of neutral tone are F0 and duration (Lin and Yan, 1980; Lin, 1983; Cao, 1986; Yang, 1989; Wang, 2004; Chen and Xu, 2006; Li and Fan, 2015), with F0 being more important than duration (Cao, 1986; Wang, 2004; Li and Fan, 2015; Li, 2017). Spectral tilt is a reliable cue, but it is less important than duration (Zhong et al., 2001). Intensity is not reliable (Lin and Yan, 1980; Lin, 1983). The same acoustic correlates are found for lexical stress in stress language, with duration being the most reliable cue for lexical stress in Dutch (Sluijter and van Heuven, 1995, 1996; van Heuven and de Jonge, 2011).

Previous research has revealed inconsistencies regarding how infants perceive lexical tones and lexical stress early in life. Some studies found supportive evidence for the perceptual reorganization of lexical tones, which occurred around 9 months. For example, prior to 6 months, both tone- and non-tone-language infants can discriminate lexical tones. By around 9 months, non-tone-language infants’ sensitivity to lexical tones declines, whereas no such decline is observed among tone-language infants (Mattock and Burnham, 2006; Mattock et al., 2008). Some other studies, however, reported different results. For instance, in Liu and Kager (2014), 5- to 18-month-old Dutch infants showed continuous discrimination of Mandarin T1–T4 contrast. But when the phonetic distance between T1 and T4 was reduced, the infants no longer demonstrated discrimination. In Chen and Kager (2016), 4-month-old Dutch infants failed to discriminate a non-salient Mandarin tonal contrast (T2–T3), yet 6- and 12-month-old infants succeeded. Infants may not be born with the ability to discriminate all the native contrasts and may especially need time to learn phonetically non-salient contrasts (Sundara et al., 2006; Narayan et al., 2010). For lexical tones, Shi (2010) discovered that Mandarin infants were only able to categorize phonetically variable lexical tones gradually after 8 months. In Tsao (2008), 12-month-old Mandarin infants discriminated T1–T3 better than T2–T3/T2–T4 contrasts. Taken together, early discrimination of lexical tones appears to exhibit a complex developmental pattern, where successful discrimination might relate to the phonetic salience of particular tonal contrasts.

In terms of lexical stress, in studies supporting perceptual reorganization, infants’ stress perception appears to shift from universal discrimination to their native language at 9 months of age (Sansavini et al., 1997; van Ooijen et al., 1997; Hohle et al., 2009; Skoruppa et al., 2009, 2013). For example, newborn French infants could discriminate stress-initial and stress-final words (Sansavini et al., 1997), while 9-month-old French infants failed to discriminate stress contrast at a phonological level. Hence, French infants adapted their stress perception to their native language by 9 months. Nine-month-old Spanish infants, whose native language has contrastive lexical stress, demonstrated discrimination (Skoruppa et al., 2009). In some other studies, however, the discrimination of contrastive lexical stress requires sufficient exposure to ambient input (Weber et al., 2004; Keij and Kager, 2013; Butler et al., 2015). For instance, 5-month-old German infants could discriminate between stress-initial and stress-final pseudowords, yet 4-month-old German infants could not (Weber et al., 2004). In summary, attunement seems flexible in early language perception. It might be modulated by ambient language input for lexical tone and lexical stress. For lexical tone, participants’ discrimination could be related to the acoustic salience of particular stimuli.

Besides acoustic salience, the order of stimuli presentation may influence the discrimination effect as well. Perceptual asymmetry was found in previous studies on the discrimination of both segments (Polka and Werker, 1994; Polka and Bohn, 1996, 2003) and suprasegments (Weber et al., 2004, 2005; Tsao, 2008; Chen, 2013; Segal et al., 2016). In Segal et al. (2016), when discriminating between initial and final lexical stress, Hebrew infants showed better discrimination when presented with uncommonly initial stress first. German-learning infants also showed similar perceptual asymmetry when perceiving lexical stress, namely that change detection was easier for infants when trochee, the predominant stress pattern, was embedded in iambs rather than the other way around (Weber et al., 2004). For early perception of lexical tones, Mandarin infants discriminated the T1–T3 contrast better if they were presented with T1 first than the other way around (Tsao, 2008). The mechanism underlying such asymmetry is not fully understood, yet it may be related to statistical distribution in the input. When habituated to an atypical pattern in ambient input, infants may consolidate such a pattern in representation and subsequently discriminate the frequent pattern in the input from the infrequent one. Yet if infants are habituated to the frequent pattern in the input, they might perceive the infrequent pattern as a non-prototypical realization of the frequent one.

The statistical distribution of particular phonological features in the input influences infants’ perceptions of such features. Scholars have largely agreed that infants are sensitive to statistical distribution in speech input (e.g., Saffran et al., 1996; Maye et al., 2002). Infants prefer predominant patterns to which they are exposed in their native language, and such preferences are established with accumulating exposure (Jusczyk et al., 1993). In the current study, we compared stress-language (Dutch) and tone-language (Mandarin) infants on their discrimination of canonical and neutral tones. Because neutral tone carries lexical stress and tonal information, it serves as a feasible means to investigate early attunement to lexical tone or stress as the result of ambient input. We posed the following questions in the current study: (1) whether Mandarin infants can discriminate between neutral and canonical tones, and whether such discrimination is influenced by acoustical salience of the tones; and (2) whether Dutch listeners perceive neutral tone as tonal or as lexical stress, and whether perceptual reorganization can be observed for neutral tone. We began by testing whether tone- and stress-language-speaking adults perceived neutral tone as unstressed, which served as a baseline for the subsequent infant experiments. Next, we tested 4- to 6-month-old and 10- to 12-month-old Dutch and Mandarin infants on their discrimination of Mandarin canonical–neutral tone contrast. If Dutch infants perceived the canonical–neutral tone contrast as lexical stress, we would expect successful discrimination at both ages; on the other hand, if they perceived them as tonal, discrimination may only be successful for the younger group. For Mandarin infants, we expected them to be capable of discriminating the contrasts at both ages. Considering that sequences with neutral tone occur less frequently than those involving canonical tones, it may take time for Mandarin infants to learn these contrasts. In this case, we would expect only the 10- to 12-month-old Mandarin infants to discriminate the contrasts.

## Experiment 1: Adults’ Perceptions of Neutral Tone

To understand whether Dutch adult listeners perceive neutral tone as unstressed, a discrimination task and an identification task were conducted in Experiment 1. In the discrimination task, participants were required to discriminate disyllabic sequences ending in a neutral tone from those ending in a canonical tone. If Dutch adult listeners perceived neutral tone as unstressed, they would discriminate canonical–neutral tone contrast successfully. In the identification task, participants were required to identify the position of stress in the disyllabic sequences. Because duration is the most reliable cue for lexical stress in Dutch, and neutral tone exhibits a shorter duration compared with canonical tones, we predicted that Dutch adult listeners would identify the neutral tone as unstressed. For Mandarin listeners, given T0 as a category in native phonology, we assumed they would succeed in the discrimination task and thus be able to identify the neutral tone as unstressed.

### The Discrimination Task

#### Stimuli

The pseudoword /pansan/ was selected as the tone-bearing sequence, which is a well-formed sequence phonotactically in Mandarin and Dutch. All possible tone combinations were included except T3T3, which is always produced as T2T3 due to the Mandarin tone sandhi process. In total, 19 target pseudowords were obtained, including 15 disyllabics ending with a canonical tone (4 × 4 - 1 = 15) and 4 disyllabics ending with a neutral tone (TnT0; *n* = 1, 2, 3, or 4). Another 20 tonal pairs of real words in Mandarin were added as fillers, which carried the same segments but different canonical tones, such as 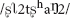 (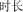, duration) vs. 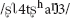 (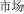, market).

All stimuli were produced by a 35-year-old male native Mandarin speaker. The speaker was born and raised in Beijing. No disorder was reported related to reading, speaking, or listening. Nineteen pseudowords were recorded along with 40 filler words in the soundproof room of the phonetics lab at the Chinese Academy of Social Sciences (CASS) using Cool Edit Pro 2.0 at a sample rate of 44,100 Hz.

#### Participants

Eighteen Mandarin adult listeners were tested, 10 males and 8 females, with an average age of 20.8 years (*SD* = 1.9). Another participant took part in the test but was excluded due to equipment failure. All participants were born and raised in Beijing, without reported hearing or speech disorders.

Eighteen Dutch adult listeners were tested, 6 males and 12 females, with an average age of 23.7 years (*SD* = 4.7). They were born and raised in the Netherlands. None of the participants had been exposed to any tone language, and no hearing or speech disorders were reported.

#### Procedures

The AX paradigm was adopted. Participants were presented with pairs of stimuli and required to indicate whether the two stimuli were the same or different. The series consisted of 30 pairs of different stimuli (AX or XA) and 19 pairs of identical stimuli (AA or XX). For each different pair, the comparison was only conducted between a sequence ending in a canonical tone and its corresponding neutral tone form. Taking /pan1san1/ as an example, its neutral tone form was “/pan1san0/”. The different pairs were “/pan1san0/ vs. /pan1san1/” and “/pan1san1/ vs. /pan1san0/”, and the identical pairs were “/pan1san1/ vs. /pan1san1/” and “/pan1san0/ vs. /pan1san0/”. Another 80 pairs of fillers included different pairs such as “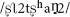 (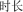, duration) vs. 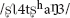 (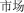, market)” and identical pairs such as “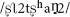 (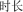, duration) vs. 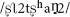 (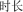, duration).”

A practice phase preceded the experiment. Seven pairs of stimuli were used to familiarize participants with the procedure. Each trial started with a fixation cross, followed by two audio stimuli with an inter-stimulus interval of 200 ms. When the audio stimuli concluded, two buttons were shown on the screen, labeled as “Same (F)” and “Different (J).” Participants provided their response by pressing either “F (Same)” or “J (Different)” on the keyboard. The next trial started automatically after the participant had responded. The inter-trial interval was 500 ms. ZEP was used to control the procedures, randomize stimuli, and collect participants’ responses (Veenker, 2013).

#### Results

**FIGURE 2:**
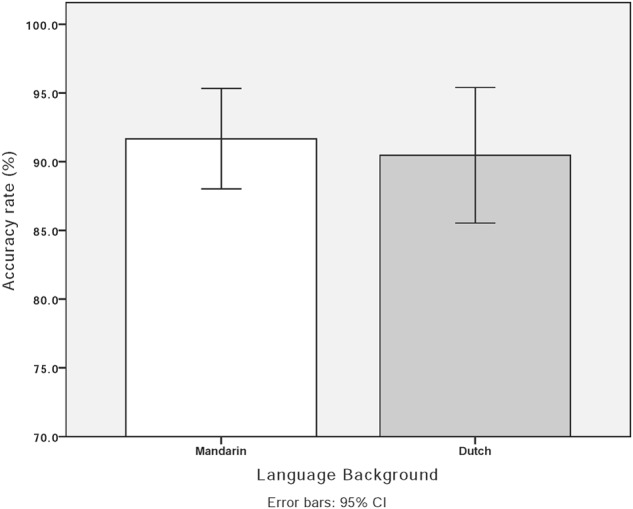
Accuracy rate in the AX discrimination task by Mandarin and Dutch adult listeners.

The accuracy rate was calculated by dividing the number of correct responses by the number of total trials for each participant. For identical pairs, the accuracy rate for Mandarin listeners was 93% (*SD* = 1.24) and 97.1% for Dutch listeners (*SD* = 0.55). When discriminating different stimuli pairs, the accuracy rate for Mandarin listeners was 91.7% (*SD* = 0.74) and 90.5% (*SD* = 0.99) for Dutch listeners. **Figure 2** illustrates the accuracy rates of Mandarin and Dutch adult listeners. To better understand participants’ sensitivity to the canonical–neutral contrast, *d*-prime (*d*′) was calculated. An independent *t*-test was conducted using *d*-prime with the language group as the independent variable. No difference was found between Dutch and Mandarin adult listeners [*t*(34) = -0.57, *p* > 0.05].

Both Dutch and Mandarin adult listeners could discriminate neutral and canonical tones. To further investigate whether they perceived the neutral tone as unstressed, we conducted the following identification task.

### The Identification Task

#### Stimuli

The stimuli in the discrimination task were used in the identification task. Participants were asked to indicate the stress position in disyllabic sequences ending in a neutral tone as well as those ending in a canonical tone.

To ensure that the Dutch participants understood the task, we used Dutch lexical stress minimal pairs as practice stimuli. We selected three minimal pairs in Dutch: “kaNON – KAnon,” “voorNAAM – VOORnaam,” and “SERvisch – serVIES” (capital letters denote the stressed syllables) (Cutler and van Donselaar, 2001). A female Dutch native speaker produced all minimal pairs by reading each pair twice. All recordings were completed in the phonetics lab at Utrecht University using Audacity at a sample rate of 32,000 Hz. Five native Dutch-speaking phoneticians selected the most naturally produced pairs for the identification task.

#### Participants

Another 15 Mandarin listeners different from those in the discrimination task were tested, 5 males and 10 females (mean age = 21.5 years; *SD* = 0.58). All Mandarin listeners were born and raised in Beijing, and no hearing disorders were reported. Another two participants were tested but excluded due to dyslexia (*N* = 1) and a reported background in phonetics (*N* = 1).

Another 14 Dutch listeners were tested, 6 males and 8 females (mean age = 25.9 years; *SD* = 8.4). All participants were born and raised in the Netherlands and reported no exposure to tone languages. No hearing disorders were reported. Another 6 Dutch listeners were tested but excluded for failing to identify the lexical stress for Dutch stress minimal pairs.

#### Procedures

A forced-choice procedure was adopted. The experiment was preceded by a practice phase in which 12 trials were used to familiarize the participants with the task. In each trial, Dutch listeners heard one word of the Dutch stress minimal pairs, such as “kaNON.” The participants were required to identify the position of lexical stress. They were asked to give their response by clicking one of the buttons labeled “Initial (Strong-weak, Sw)”, “Equal”, or “Final (weak-Strong, wS)”. Each word of the Dutch stress minimal pairs was repeated twice. Mandarin listeners heard 12 trials. They were presented with Mandarin disyllabic sequences, such as “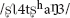 (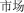, market),” and were required to indicate whether the word had initial, equal, or final stress by clicking the corresponding button.

Each trial in the test phase began with a fixation cross, after which an audio stimulus was presented. Participants were required to give their responses by clicking one of the buttons labeled “Initial,” “Equal,” or “Final.” Another two buttons were below these, labeled “Repeat” and “Next.” Participants were allowed to listen to the stimulus again by clicking “Repeat.” By clicking the “Next” button, participants submitted their options and activated the next trial. ZEP was used to control the procedures, randomize stimuli, and collect participants’ responses (Veenker, 2013).

#### Results

The accuracy rate in the practice phase was calculated for Dutch listeners. Only data from participants with accuracy rates over 80% in the practice phase were submitted for further analysis, including 6 males and 8 females (mean age = 25.9 years; *SD* = 8.4).

For each option, we calculated selection percentages under different tonal conditions, including neutral tone, canonical tone, and each individual tonal combination. Taking “Initial” as an example, the percentage was calculated by dividing the number of responses indicating “Initial” by the total number of responses. A Chi-square test was conducted to access whether participants’ responses depended on their language background when presented with disyllabic sequences ending in a neutral tone. The results were not significant, χ^2^(2) = 0.63, *p* > 0.05. Hence, when presented with disyllabic sequences ending in a neutral tone, no difference appeared between Mandarin and Dutch listeners. Both groups predominantly selected “Initial” for sequences ending in a neutral tone. However, for sequences with two canonical tones, a significant relationship emerged between participants’ responses and language background [χ^2^(2) = 24.15, *p* < 0.01, φ = 0.24]. Mandarin listeners tended to identify stimuli as “Equal” (56.9%), while 23.8% of Dutch listeners selected “Initial,” 35.2% chose “Equal,” and 41% chose “Final.” Participants’ responses across the three options are listed in **Table 1**.

**Table 1:** The selection percentage of “Initial,” “Equal,” and “Final” in neutral/canonical tone combinations for Dutch and Mandarin listeners.

Combinations	Language	Initial (%)	Equal (%)	Final (%)
Neutral tone	Mandarin	85	13.3	1.7
	Dutch	80.4	16.1	3.6
Canonical tones	Mandarin	10.7	56.9	32.4
	Dutch	23.8	35.2	41

**FIGURE 3:**
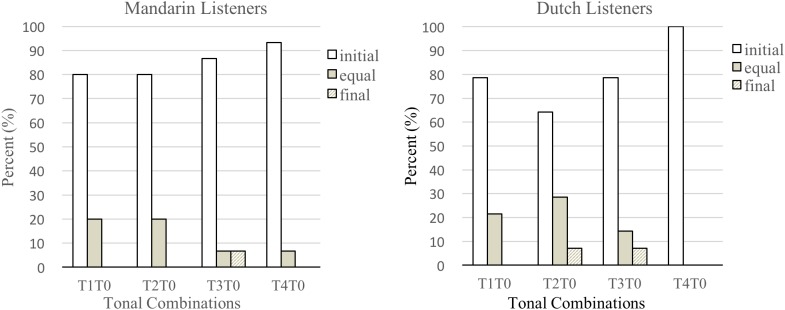
Mandarin and Dutch adult listeners’ identification of neutral tone combinations.

For particular tonal combinations ending in a neutral tone, 80% of Mandarin listeners identified T1T0 as “Sw (Initial).” Percentages for other sequences involving neutral tone were T2T0 (80%), T3T0 (86.7%), and T4T0 (93.3%). Seventy-eight percent of Dutch listeners identified T1T0 as “Sw.” The percentages for other sequences involving neutral tone were T2T0 (64.3%), T3T0 (78.6%), and T4T0 (100%). All percentages are plotted in **Figure 3**.

**FIGURE 4:**
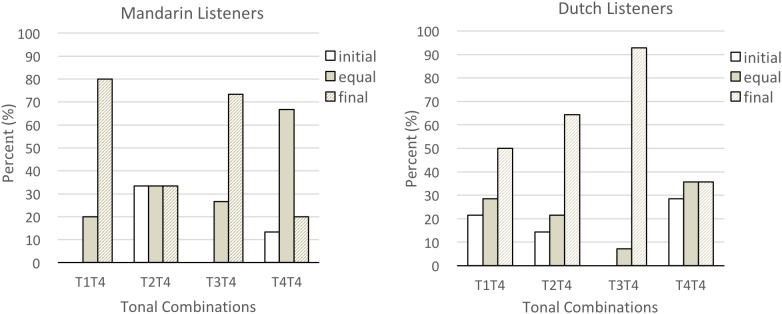
Mandarin and Dutch listeners’ identification of sequences ending in T4.

To select the tonal contrast for the subsequent infant experiment, we also analyzed participants’ identification of disyllabic sequences with two canonical tones. The ending T4 was predominantly perceived as stressed. For Mandarin listeners, the percentages selecting “wS (final)” were 80% for T1T4, 33.3% (T2T4), 73.3% (T3T4), and 20% (T4T4). For Dutch listeners, the percentages selecting “wS (final)” were 50% for T1T4, 64.3% (T2T4), 92.9% (T3T4), and 35.7% (T4T4) (see **Figure 4**). Except for tonal combinations ending with T4, Mandarin listeners tended to perceive disyllabic sequences with two canonical tones as being of “equal weight.”

### Discussion

Dutch (stress language) adult listeners could discriminate disyllabic sequences ending in a neutral tone from those ending in a canonical tone. In addition, Mandarin and Dutch listeners identified a neutral tone as unstressed. Overall, Mandarin listeners tended to perceive sequences with two canonical tones as having equal stress, consistent with the fact that Mandarin generally lacks word stress.

Because neutral tone contains simultaneous tonal and stress information, the following notions warrant investigation: (1) how infants process neutral tone, and whether younger infants (4- to 6-month-olds) and elder infants (10- to 12-month-olds) respond differently; and (2) whether ambient language input influences infants’ perceptions. Specifically, we asked the following questions in Experiment 2: Can Mandarin- and Dutch-learning infants discriminate neutral tones from canonical tones? Do stress-language-learning infants perceive neutral tone as unstressed? Given that 10- to 12-month-old infants are attuned to their native language, would 4- to 6-month-old infants respond differently from 10- to 12-month-olds? To this end, we tested 4- to 6-month-old and 10- to 12-month-old Dutch and Mandarin infants on their discrimination of disyllabic sequences that either ended in a canonical (T4) or a neutral tone (T0). Since neutral tone is a native phonological category for Mandarin-learning infants, we predicted they would be able to discriminate between neutral tone and canonical tone throughout the 1st year of life. No difference was expected between 4- to 6-month-old and 10- to 12-month-old Mandarin infants. Given that Dutch adults were able to identify neutral tone as unstressed, if they processed neutral tone as lexical stress, they would show continuous discrimination throughout the 1st year of life. Thus, no difference was expected between 4- to 6-month-old infants and 10- to 12-month-old Dutch infants. However, if they processed the difference between T1T4 and T1T0 as tonal information instead of lexical stress, 10- to 12-month-old Dutch infants would fail to distinguish the tonal contrast, while 4- to 6-month-old Dutch infants were expected to discriminate the contrast successfully.

## Experiment 2: Infants’ Discrimination of Canonical Tone and Neutral Tone

### Stimuli

The pseudoword /pansan/ was used as stimuli. In the above discrimination and identification tasks, Dutch listeners identified T1T4 as “wS (final)” and T1T0 as “Sw (initial),” respectively. Hence, we selected these two sequences as stimuli for the infant experiment.

A 32-year-old female Mandarin native speaker produced neutral tone (/pan1san0/) and canonical tone (/pan1san4/) in infant-directed speech (IDS). Each stimulus was produced 20 times. Recordings were completed in the soundproof room of the phonetics lab at CASS using Cool Edit Pro 2.0 at a sample rate of 16,000 Hz. Another five Mandarin native speakers judged the naturalness of recordings on a continuum from 1 (extremely unnatural) to 5 (very natural). Two phoneticians selected the six most natural tokens for T1T0 (/pan1san0/) and six most natural tokens for T1T4 (/pan1san4/).

**FIGURE 5:**
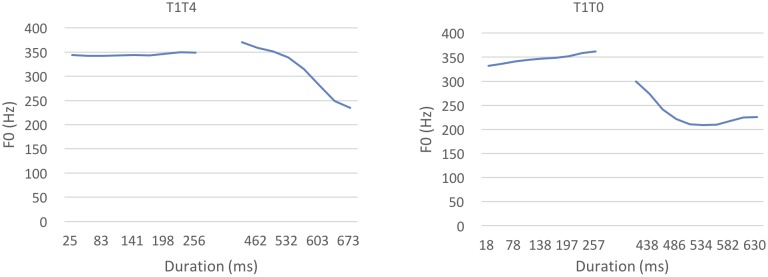
Average F0 contours of T1T4 and T1T0.

For the canonical tone sequence /pan1san4/, the average duration of the first syllable was 259.7 ms (*SD* = 10.8), and the average duration of the second syllable was 316.8 ms (*SD* = 23.4). For the neutral tone sequence /pan1san0/, the first syllable was 269.2 ms on average (*SD* = 11.8), and the second syllable was 216 ms on average (*SD* = 41.8). For F0 contour, 10 F0 points were extracted along the F0 contour using Praat (Boersma and Weenink, 2013). For T1T4 combinations, the maximal F0 value of T4 was 307.4 Hz, and its minimal F0 value was 234.3 Hz with a range of 136.08 Hz. For T1T0 combinations, the maximal F0 value of T0 was 303.9 Hz, and its minimal F0 value was 218.5 Hz with a range of 85.4 Hz. The averaged F0 contours are shown in **Figure 5**, where T4 (high-falling) and T0 (mid-falling) exhibited similar falling contours.

### Participants

Fifty-two Dutch-learning infants were tested: 23 were between 4–6 months old (mean age = 4;18, *SD* = 0.7, 11 males and 12 females), and 29 were between 10–12 months old (mean age = 11;3, *SD* = 0.8, 19 males and 10 females). Another 20 infants were tested but excluded due to fussiness (*N* = 6), parental intervention (*N* = 1), and not being habituated (*N* = 13). All Dutch-learning infants were born and raised in Dutch-speaking families where Dutch was the only language in use. All parents reported normal hearing of the infants. Dutch-learning infants were tested in the infant lab at Utrecht University (UU).

Among the 24 Mandarin-learning infants tested, 8 were between 4–6 months old (mean age = 5;9, *SD* = 0.9, 4 males and 4 females), and 16 were between 10–12 months old (mean age = 11;21, *SD* = 1.1, 6 males and 10 females). Another 16 infants were tested but excluded due to fussiness (*N* = 5), parental intervention (*N* = 1), not being habituated (*N* = 5), equipment failure (*N* = 3), dialect interference in the input (*N* = 1), and being a preterm infant (*N* = 1). All Mandarin-learning infants were born and raised in Mandarin-speaking families where Mandarin was the only language in use. All parents reported normal hearing of the infants. Mandarin-learning infants were tested in the infant lab at CASS, Beijing.

### Procedures

A visual fixation procedure was adopted. During the experiment, a parent sat in a chair in the test cabin listening to music played through headphones to prevent possible intervention. The infant sat on his/her parent’s lap, facing the screen in the front of the test cabin. The screen was one meter away from the infant, and the visual stimuli was played on the screen during the experiment. Two loudspeakers were situated on both sides of the cabin along with a hidden video camera above the screen. The camera was connected to a screen on the control desk, which was used to observe infants’ responses to stimuli in real time. The control desk was in a separate room next to the test cabin.

There were four phases: pre-test, habituation, test, and post-test. The pre- and post-test were used to test infants’ general attention. In the habituation phase, infants were habituated to either canonical tone (T1T4, /pan1san4/) or neutral tone (T1T0, /pan1san0/). In the test phase, canonical tone and neutral tone sequences alternated between trials. In the habituation phase, three tokens from each category were used to habituate Mandarin- and Dutch-learning infants. Chen and Kager (2016) reported that 4-month-old Dutch infants could not normalize multiple tokens of tonal contrast. Thus, only another one token was used in the test phase for Dutch infants. We used another three tokens in the test phase for Mandarin infants.

Each trial started with an attention-getter. Once the infant looked at the screen, the attention-getter faded out, and the visual stimuli and audio stimuli were played. The infant’s looking time and non-looking time were recorded by the experimenter on the control computer. When the average looking time of three consecutive trials was shorter than 50% of the average looking time of the first three trials, the habituation criterion was met, and the test phase started automatically. The habituation phase had a maximum of 16 trials.

The test phase consisted of four trials, two of which were identical to the habituated tone sequences (same trials). The other two trials were the tone sequences that were not used in the habituation phase (novel trials). The same trials and novel trials alternated. Infants’ looking time during the same trials and novel trials were recorded by the control computer. If they were able to discriminate the tonal sequences, then their looking time during the novel trials would presumably be longer than during the same trials.

### Results

To correct for skewness, the raw looking time was logarithmically transformed. Dutch- and Mandarin-learning infants were divided into two age groups: 4- to 6-month-olds and 10- to 12-month-olds. For each age group, the log-transformed looking time (LogLT) of the same trials and of the novel trials were compared.

#### Dutch-Learning Infants

**FIGURE 6:**
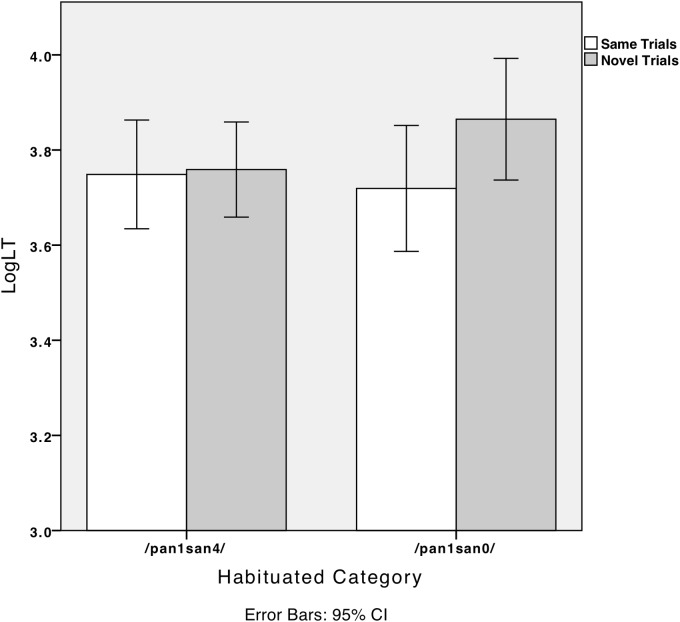
Dutch-learning infants’ log-transformed looking time (LogLT) in “same trials” and “novel trials” when habituated to canonical tone (/pan1san4/) or neutral tone (/pan1san0/).

We conducted a 2 (trial type: same/novel) × 2 (habituated category: neutral tone/canonical tone) × 2 (age group: 4- to 6-month-olds/10- to 12-month-olds) mixed effect ANOVA. Trial type was the within-subject factor. The between-subject factors were habituated category and age group. Trial type showed a main effect [*F*(1,48) = 6.3, *p* < 0.05, ${\text{η}}_{\text{p}}^{2}$ = 0.12] with the looking time in the novel trials being significantly longer than in the same trials (*p* < 0.05). Age group had no main effect [*F*(1,48) = 0.22, *p* > 0.05], nor did the habituated category [*F*(1,48) = 0.39, *p* > 0.05]. There was significant interaction between trial type and the habituated category, *F*(1,48) = 4.5, *p* < 0.05, ${\text{η}}_{\text{p}}^{2}$ = 0.09. When infants were habituated to T1T0, the looking time in the novel trials was significantly longer than in the same trials, *t*(23) = -2.58, *p* < 0.05. However, when infants were habituated to T1T4, there was no difference between the same trials and the novel trials, *t*(27) = -0.28, *p* > 0.05. **Figure 6** plots the infants’ looking times separated by habituated tone. Neither the interaction between trial type and age group [*F*(1,48) = 1.80, *p* > 0.05] nor the three-way interaction among trial type, habituated category, and age group [*F*(1,48) = 0.04, *p* > 0.05] was significant.

**FIGURE 7:**
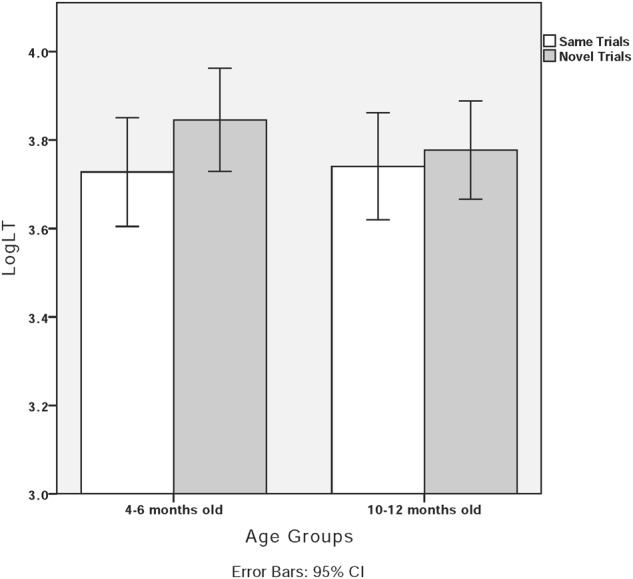
Dutch-learning infants’ log-transformed looking time (LogLT) in “same trials” and “novel trials” for each age group (T1T4 vs. T1T0).

Although there was no interaction between trial type and age group, to better capture the perception pattern within each age group, we looked at the data for 4- to 6-month-old and 10- to 12-month-old infants separately. Dutch 4- to 6-month-old infants looked longer at the novel trials (average LogLT = 3.85, *SD* = 0.27) than the same trials (average LogLT = 3.73, *SD* = 0.28), *t*(22) = -2.26, *p* < 0.05. No difference was found between the same trials (average LogLT = 3.74, *SD* = 0.32) and novel trials (average LogLT = 3.78, *SD* = 0.29) for 10- to 12-month-old infants, *t*(22) = -0.84, *p* > 0.05. These findings suggest that 4- to 6-month-old infants might be more sensitive to neutral–canonical contrast than 10- to 12-month-old infants. **Figure 7** plots the infants’ log-transformed looking time in the same and novel trials for each age group.

#### Mandarin-Learning Infants

We conducted the same 2 (trial type: same/novel) × 2 (habituated category: neutral tone/canonical tone) × 2 (age group: 4- to 6-month-olds/10- to 12-month-olds) mixed effect ANOVA on the data obtained from Mandarin infants. Trial type failed to show a main effect [*F*(1,20) = 0.03, *p* > 0.05]. Habituated category did not show a main effect, *F*(1,20) = 0.003, *p* > 0.05. Age group did not show significant main effect either, *F*(1,20) = 0.10, *p* > 0.05. The interaction between trial type and age group was marginally significant, *F*(1,20) = 3.58, *p* = 0.07, ${\text{η}}_{\text{p}}^{2}$ = 0.15. No interaction was found between trial type and habituated category [*F*(1,20) = 0.09, *p* > 0.05] or among trial type, habituated category, and age group [*F*(1,20) = 0.31, *p* > 0.05].

**FIGURE 8:**
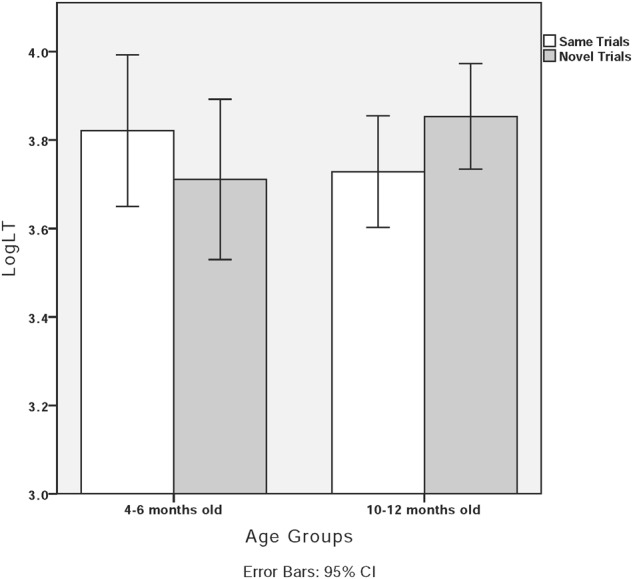
Mandarin-learning infants’ log-transformed looking time (LogLT) in “same trials” and “novel trials” for each age group (T1T4 vs. T1T0).

Because we found a marginally significant interaction between age group and trial type, we conducted paired *t*-tests with 4- to 6-month-old and 10- to 12-month-old infants separately. The results showed no difference between the same trials and novel trials for the 4- to 6-month-old infants [*t*(7) = 1.41, *p* > 0.05] or the 10- to 12-month-old infants [*t*(15) = -1.61, *p* > 0.05]. **Figure 8** shows the looking time in the same and novel trials for 4- to 6-month-old and 10- to 12-month-old infants.

### Discussion

In the present experiment, we tested Dutch and Mandarin infants’ discrimination of canonical (T1T4, /pan1san4/) and neutral tone (T1T0, /pan1san0/). Regardless of age, Dutch-learning infants were able to discriminate T1T4–T1T0 contrast. Given that Dutch adults perceived T1T4 and T1T0 as “wS” and “Sw,” respectively, Dutch infants likely processed neutral–canonical tone contrast as lexical stress instead of tonal information. Perceptual asymmetry was found for Dutch infants: those who were habituated to T1T0 discriminated T1T4–T1T0 contrast; those habituated to T1T4 did not. For languages with a predominant initial stress pattern, such as English, German, and Dutch, infants demonstrated an initial stress preference (Fikkert, 1993; Jusczyk et al., 1993; Friederici et al., 2007). For Dutch infants, the trochaic pattern, which is T1T0 in the present study, may be more salient to perceive than the less frequent iambic T1T4 pattern. When presented with T1T0 first, it might be easier for infants to consolidate the representation of the salient pattern, which allows for later successful discrimination. When presented with the iambic pattern, infants may accept such a pattern as a non-prototypical realization of the trochaic pattern.

Both the 4- to 6-month-old and the 10- to 12-month-old Mandarin infants unexpectedly failed to discriminate between the neutral–canonical tone contrast, and no perceptual asymmetry emerged. In previous studies, acoustic salience influenced participants’ discrimination (Tsao, 2008; Liu and Kager, 2014; Chen and Kager, 2016). With regard to T1T4 and T1T0, T4 and T0 showed a similar falling pitch contour and similar register (Li, 2017). It is possible that T0 and T4 were not distinctive enough for Mandarin infants to discriminate them. According to the assumption of perceptual assimilation model (Best, 1994), Mandarin infants might perceive T1T0 as a realization of T1T4 and vice versa. As such, the failed discrimination could have been caused by the acoustic similarities between T1T4 and T1T0.

**FIGURE 9:**
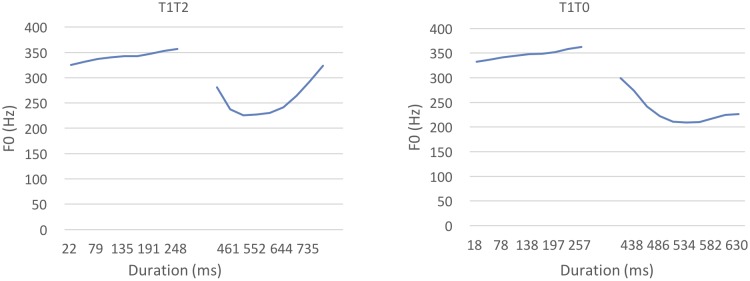
Average F0 contours of T1T2 and T1T0.

Therefore, we tested Mandarin infants in Experiment 3 using a more salient canonical–neutral tone contrast, the T1T2–T1T0 contrast. Unlike the T1T4 and T1T0 contrast, where both T4 and T0 exhibit a falling contour, in the T1T2 and T1T0 contrast, T2 exhibits a rising contour and T0 exhibits a falling contour (see **Figure 9**). Compared with T4, the pitch contour of T2 is more different from T0 (Li, 2017). If the phonetic similarity between T1T4 and T1T0 indeed hindered Mandarin infants’ discrimination, the more salient acoustic difference would be expected to allow Mandarin infants to discriminate T1T2 and T1T0.

## Experiment 3: Mandarin Infants’ Discrimination of T1T2 and T1T0

### Stimuli

The pseudoword /pansan/ was also used as stimuli. Infants were tested on their discrimination of T1T2 and T1T0. T1T2 and T1T0 carried saliently different pitch contours: T1T2 was “high-level + mid-rising,” but T1T0 was “high-level + mid-falling.” As T0 was realized in a shortened duration, T1T0 exhibited a “long-short” duration pattern.

The same female Mandarin native speaker produced /pan1san2/ in IDS 20 times. Recordings were completed in the soundproof room of the phonetics lab at CASS, using Cool Edit Pro 2.0 at a sample rate of 16,000 Hz.

Another five native Mandarin speakers judged the naturalness of the recordings on a continuum from 1 (extremely unnatural) to 5 (very natural). Two phoneticians selected the six most natural tokens of /pan1san2/. The six tokens of neutral tone (/pan1san0/), which were used in Experiment 2, were also used in the present experiment. In the six tokens of each category, three were used in the habituation phase, and another three were used in the test phase.

For the canonical tone sequence of T1T2 (/pan1san2/), the average duration of the first syllable was 253.5 ms (*SD* = 12), and the second syllable was 411.3 ms (*SD* = 15.7). For the neutral tone sequence of T1T0 (/pan1san0/), the average duration of the first syllable was 269.2 ms (*SD* = 11.8), and the second syllable was 216 ms (*SD* = 41.8). For tonal contours, 10 F0 values were extracted on the F0 contour of each tone using Praat (Boersma and Weenink, 2013). For the T1T2 sequences, the maximal F0 value of T2 was 323.7 Hz, and its minimal F0 value was 226 Hz with a range of 97.7 Hz. For T1T0 sequences, as used in Experiment 2, the maximal of T0 was 303.9 Hz, and its minimal F0 value was 218.5 Hz with a range of 85.4 Hz. The average F0 contours are presented in **Figure 9**.

### Participants

Thirty-five Mandarin-learning infants different from those in Experiment 2 were tested. Eight of them were 4–6 months old (mean age = 5;16, *SD* = 0.7, 4 males and 4 females) and 27 were 10–12 months old (mean age = 10;9, *SD* = 2.8, 16 males and 11 females). Another 34 infants were tested but later excluded due to fussiness (*N* = 10), parental intervention (*N* = 7), dialect interference in the input (*N* = 6), not being habituated (*N* = 4), equipment failure (*N* = 4), and experimenter error (*N* = 3). All Mandarin-learning infants were born and raised in Mandarin-speaking families where Mandarin was the only language in use. All parents reported normal hearing of the infants. Mandarin-learning infants were tested in the infant lab at CASS, Beijing.

### Procedures

The experimental procedures were the same as in Experiment 2 (see section “Procedures” under the section “Experiment 2: Infants’ Discrimination of Canonical Tone and Neutral Tone”). All experiments were completed in the infant lab at CASS, Beijing.

### Results

To correct for skewness, the raw looking time was logarithmically transformed. Mandarin-learning infants were divided into two age groups: 4–6 months old and 10–12 months old. For each age group, the LogLT in the same trials and novel trials were compared.

We conducted the same analysis as in Experiment 2: a 2 (trial type: same/novel) × 2 (habituated category: neutral tone/canonical tone) × 2 (age group: 4–6/10–12 months old) mixed effect ANOVA was conducted. Trial type served as the within-subject factor. The between-subject factors were habituated category and age group. Trial type showed no main effect [*F*(1,31) = 1.68, *p* > 0.05], nor did habituated category [*F*(1,31) = 0.03, *p* > 0.05] or age group [*F*(1,31) = 0.42, *p* > 0.05]. There was also no interaction between trial type and age group, *F*(1,31) = 1.18, *p* > 0.05. The interaction between trial type and habituated category, however, was significant, *F*(1,31) = 4.53, *p* < 0.05, ${\text{η}}_{\text{p}}^{2}$ = 0.13. No significant interaction was found among trial type, habituated category, and age group, *F*(1,31) = 0.22, *p* > 0.05.

**FIGURE 10:**
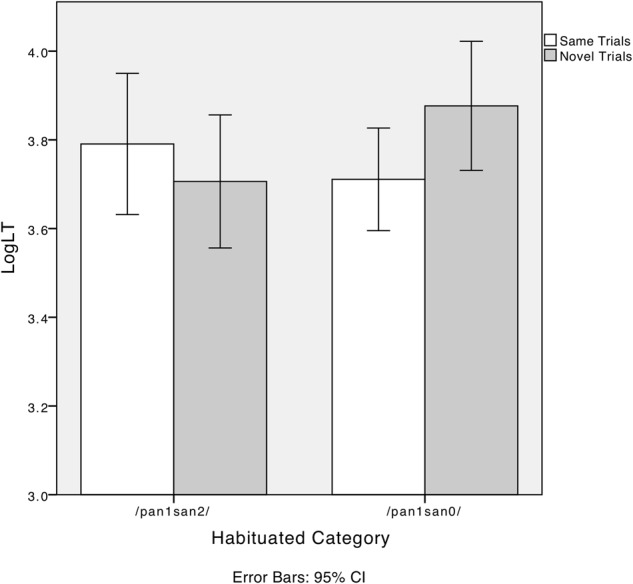
Mandarin infants’ log-transformed looking time (LogLT) in “same trials” and “novel trials” when habituated to canonical tone (/pan1san2/) or neutral tone (/pan1san0/).

We further split the data according to the habituated category to examine the interaction between trial type and habituated category. Paired *t*-tests were conducted to compare infants’ looking time in the same trials and novel trials. When infants were habituated to canonical tones (T1T2, /pan1san2/), there was no difference between looking time in the same trials and novel trials [*t*(14) = 1.5, *p* > 0.05]. However, when infants were habituated to neutral tone (T1T0, /pan1san0/), their looking time in the novel trials was significantly longer than in the same trials [*t*(19) = -2.51, *p* < 0.05]. **Figure 10** shows the interaction between trial type and habituated category.

### Discussion

When presented with an acoustically salient contrast (T1T2–T1T0), neither the 4- to 6-month-old infants nor the 10- to 12-month-old infants showed a discrimination effect. Despite the fact that neutral and canonical tones are contrastive phonetically and phonologically, Mandarin-learning infants did not seem to discriminate the neutral–canonical tone contrast during their 1st year of life.

Nevertheless, when discriminating the T1T2 and T1T0 contrast, perceptual asymmetry was evident. When infants were habituated to the sequence of T1T0 (/pan1san0/), Mandarin infants discriminated neutral (T1T0, /pan1san0/) and canonical tones (T1T2, /pan1san2/) successfully. But when they were habituated to the sequence of T1T2 (/pan1san2/), they did not show discrimination. The directional asymmetry might be related to the statistical distribution of tonal patterns in Mandarin, where canonical tones are used to distinguish lexical meanings and are therefore more common than neutral tones. About 3.8% of Mandarin vocabulary involves neutral tones (Zhu, 2009). Thus, habituating infants to the uncommon T1T0 may allow them to develop a representation of neutral tone, which would allow for later discrimination between T1T0 and T1T2. However, once habituated to the common T1T2, the uncommon T1T0 might be processed as a realization of T1T2.

## General Discussion

In the present study, we investigated the perception of neutral tone for tone- and non-tone (stress)- language listeners, both adults and infants. In Experiment 1, a discrimination task and an identification task were conducted for Mandarin and Dutch adult listeners. The results showed that Dutch adult listeners were able to discriminate between Mandarin neutral tones and canonical tones. In addition, Mandarin and Dutch listeners both identified neutral tones as unstressed. When presented with disyllabic sequences ending in a canonical tone, Mandarin listeners tended to identify the two syllables in the sequences as having “equal” stress, consistent with the claim that Mandarin does not have word level stress except for neutral tones.

In Experiments 2 and 3, we tested infants’ discrimination between neutral tone and canonical tone contrast using the visual fixation paradigm. In Experiment 2, Mandarin and Dutch infants were tested on the discrimination of a non-salient neutral–canonical tone contrast, namely T1T4 and T1T0. Results showed that Dutch infants could discriminate between neutral tones and canonical tones regardless of age. Because Dutch infants discriminated the contrast continuously, they might discriminate neutral–canonical contrast as lexical stress contrast, which exists in their native language. Perceptual asymmetry was found for Dutch infants: when they were habituated to the T1T0 sequence (/pan1san0/), they discriminated canonical–neutral contrast; when they were habituated to the T1T4 sequence, however, they could not discriminate. Trochee is the predominant stress pattern in Dutch (van Heuven and Hagman, 1988; Leyden and van Heuven, 1996). For Dutch infants, the trochaic pattern (T1T0) may be more salient than the less frequent iambic pattern (T1T4). When presented with T1T0 first, it might be easier for the infants to consolidate the representation of the salient pattern, which allows for successful discrimination later. When presented with the iambic pattern, infants may accept the pattern as a non-prototypical realization of the trochaic pattern. Unexpectedly, however, neither the 4- to 6-month-old nor the 10- to 12-month-old Mandarin infants discriminated T1T4–T1T0 contrast. In previous studies, tonal discrimination was related to acoustic salience (Tsao, 2008; Liu and Kager, 2014; Chen and Kager, 2016). Both T4 (high-falling tone) and T0 (mid-falling tone) exhibited a falling tonal contour with a similar register. Even though Mandarin infants could form a prototype of falling tone through intensive habituation, they may perceive T4 and T0 as two realizations of the same tonal category.

To this end, a more salient contrast was used as stimuli in Experiment 3: T1T2 (/pan1san2/) and T1T0 (/pan1san0/). Compared with T4, the difference between T2 and T0 was larger in tonal contour with contradicting pitch movement directions (Li, 2017). The T1T2 is “high-level + mid-rising,” and the T1T0 is “high-level + mid-falling.” Although no discrimination was found overall, we found perceptual asymmetry to be similar to the Dutch infants in Experiment 2: when infants were habituated to T1T0 (/pan1san0/), they discriminated neutral–canonical tone contrast successfully; when habituated to T1T2 (/pan1san2/), infants failed to discriminate the contrast. Taken together, acoustic salience appeared to affect infants’ discrimination. Perceptual asymmetry emerged when discriminating salient contrast. Directional asymmetry might reflect the statistical distribution of the tonal pattern in Mandarin. Canonical tones are more common in Mandarin, and neutral tones are more restricted in distribution. When habituated to the uncommon T1T0, it is likely that infants could form a new representation for neutral tone, thereby facilitating discrimination of the neutral-canonical tone contrast and leading to better discrimination.

Infants’ different responses and perceptual asymmetry in Experiments 2 and 3 may reflect properties of their native languages, such as the statistical distribution of tonal/stress pattern in the input. In Dutch, duration is the most reliable cue for lexical stress. Like native Dutch adults, Dutch infants may perceive the T1T0–T1T4 contrast as lexical stress, where T4 carries a longer duration than T0. In Mandarin, however, lexical meanings are distinguished by pitch variations. The failed discrimination between T1T0 and T1T4 might reflect the fact that both T0 (mid-falling tone) and T4 (high-falling tone) were perceived as realizations of the same falling tone. It seems that 10- to 12-month-old infants already weigh phonetic cues according to their distribution in the input (Jusczyk et al., 1993).

Based on results from the infant experiments, one could conclude that Mandarin infants in the 1st year of life cannot discriminate between neutral tone and canonical tone contrast, although in Mandarin, neutral tones contrast with canonical tones phonetically and phonologically. Acoustic salience may also affect infants’ discrimination. Mandarin infants showed perceptual asymmetry when presented with a salient contrast but not when presented with a non-salient contrast.

Particular neutral tone types may also influence infants’ discrimination. In the present study, the neutral tone in the lexeme type carried the pattern of “XY,” where X and Y stand for two different syllables. Under this condition, infants could only rely on phonetic cues to discriminate neutral and canonical tones without access to any morphological information. But in other contexts in which neutral tones are used, such as in reduplication and affixation, infants could predict neutral tones by using morphological cues, including the pattern of “XX” or “
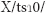
” in which the second syllable is uttered in a neutral tone. Thus, the lexical neutral tone in the form of “XY” (Y can be said in either a neutral tone or a canonical tone, leading to different lexical meanings) might be more difficult to identify than the reduplication and affixation types. In Zhu (2002), a study of the production of neutral tones, reduplication occurred as early as 14 months but remained unstable at 24 months. Affixation and the lexeme types of neutral tones emerged at 17 months and stabilized earlier than reduplication. In the current research, we approached the discrimination of neutral tone and canonical tone contrast based mainly on phonetic cues. Reduplication and affixation types will be explored in future studies. Conceivably, infants may be able to represent and distinguish neutral tones from canonical tones in contexts where morphological markers are present.

The present study had two limitations. First, the sample size of 4- to 6-month-old Mandarin infants was small. However, previous studies reported that the discrimination performance of 10- to 12-month-old infants was better than 4- to 6-month-old infants for native contrast (Narayan et al., 2010; Shi, 2010). In this study, 10- to 12-month-old Mandarin infants were unable to discriminate the neutral tone and canonical tone contrast. Hence, we hypothesized that 4- to 6-month-old Mandarin infants would not have discriminated canonical–neutral tone contrast even if the sample size had been larger. Nevertheless, increasing the sample size would likely boost the statistical power of the results and produce an interaction between age group, trial type, and/or habituation category, rendering the developmental pattern more observable. Further testing of 4- to 6-month-olds will be conducted when practicalities allow. We invite future studies for replication, and we leave the issue open for further investigation. Second, a potential factor affecting Mandarin and Dutch infants’ perceptions is tonal variability. We used multiple tokens in the habituation for Mandarin and Dutch infants, expecting that Mandarin infants would be able to represent lexical tones phonologically rather than phonetically; however, our results did not support such a hypothesis. Dutch infants, on the other hand, were able to map variable tokens in the habituation to a single token in the test phase, suggesting to some extent that they had access to abstract representation of the disyllabic sequences. The failure of the Mandarin infants might be due to the much lower frequency of neutral tones than canonical tones in the input, which could have led to the infants’ bias of accepting the variable tokens of neutral tones as realizations of canonical tones. Future studies may use single tokens to test Mandarin infants and investigate whether they can identify the difference between canonical and neutral tones on a phonetic level.

Several issues remain crucial for future studies. First, lexical knowledge might play a role in learning neutral tones in the lexeme type, and stimuli in a particular morphological structure may help highlight the developmental changes in perceiving neutral tones. For neutral tones in the context of reduplication (e.g., 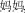, /ma1ma0/, mother)and affixation (e.g., 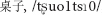, table), infants could utilize morphological cues to perceive neutral tones. For neutral tones in the lexeme type (the stimuli used in the current study) cues other than phonetic ones might be needed to perceive neutral tone. Lexical meanings may facilitate infants’ discrimination of neutral and canonical tones, but the infants tested in the current study were so young they had not yet developed sufficient knowledge of word meaning. As infants grow older, they may become capable of using word meaning to establish the representation of neutral tones. Future studies should test whether elder infants are able to use lexical meaning to discriminate between sequences ending in neutral and canonical tones.

Second, more attention should be paid to the perception of disyllabic tonal sequences. Disyllabic words are the predominant prosodic unit in Mandarin and occur more frequently than monosyllabic words (Feng, 1997; Wang, 2008). Hence, it is possible that infants learn disyllabic words holistically instead of the concatenation of individual tones, especially based on the observation that individual lexical tones are influenced by preceding and following tones due to articulation (Xu, 1997). Previous studies only used monosyllabic tones as stimuli (Mattock and Burnham, 2006; Mattock et al., 2008; Tsao, 2008; Chen, 2013; Liu and Kager, 2014; Chen and Kager, 2016), which may not reflect the actual language learning process. Knowledge of early perceptions of disyllabic canonical tone sequences among Mandarin infants will shed light on whether the acquisition of sequences involving neutral tones differ from those involving canonical tones.

In summary, a more detailed picture of neutral tone perception in future studies will emerge from the perspectives of phonetics, phonology, and word learning. Subsequent studies will provide deeper insights into discovering the process of suprasegmental information in early perception.

## Ethics Statement

This study was carried out in accordance with the recommendations of the Institute of Linguistics (CASS, China) and the Utrecht Institute of Linguistics OTS (Netherlands) with written informed consent from all adult participants/infants’ parents. All participants/infants’ parents gave written informed consent in accordance with the Declaration of Helsinki. The protocol was approved for ethics by the Institute of Linguistics (CASS, China) and the Utrecht Institute of Linguistics OTS (Netherlands).

## Author Contributions

AL, AC, and SF conceived and designed the study, and reviewed and edited the manuscript. SF performed the experiments and wrote the paper. All authors approved the manuscript.

## Conflict of Interest Statement

The authors declare that the research was conducted in the absence of any commercial or financial relationships that could be construed as a potential conflict of interest.
